# Low-dose liver CT: image quality and diagnostic accuracy of deep learning image reconstruction algorithm

**DOI:** 10.1007/s00330-023-10171-8

**Published:** 2023-09-09

**Authors:** Damiano Caruso, Domenico De Santis, Antonella Del Gaudio, Gisella Guido, Marta Zerunian, Michela Polici, Daniela Valanzuolo, Dominga Pugliese, Raffaello Persechino, Antonio Cremona, Luca Barbato, Andrea Caloisi, Elsa Iannicelli, Andrea Laghi

**Affiliations:** Department of Medical-Surgical Sciences and Translational Medicine, Radiology Unit, Sant’Andrea University Hospital, Sapienza University of Rome, Via Di Grottarossa, 1035-1039, 00189 Rome, Italy

**Keywords:** Deep learning, Iterative reconstruction, Artificial intelligence, Diagnostic accuracy, Liver

## Abstract

**Objectives:**

To perform a comprehensive within-subject image quality analysis of abdominal CT examinations reconstructed with DLIR and to evaluate diagnostic accuracy compared to the routinely applied adaptive statistical iterative reconstruction (ASiR-V) algorithm.

**Materials and methods:**

Oncologic patients were prospectively enrolled and underwent contrast-enhanced CT. Images were reconstructed with DLIR with three intensity levels of reconstruction (high, medium, and low) and ASiR-V at strength levels from 10 to 100% with a 10% interval. Three radiologists characterized the lesions and two readers assessed diagnostic accuracy and calculated signal-to-noise ratio (SNR), contrast-to-noise ratio (CNR), figure of merit (FOM), and subjective image quality, the latter with a 5-point Likert scale.

**Results:**

Fifty patients (mean age: 70 ± 10 years, 23 men) were enrolled and 130 liver lesions (105 benign lesions, 25 metastases) were identified. DLIR_H achieved the highest SNR and CNR, comparable to ASiR-V 100% (*p* ≥ .051). DLIR_M returned the highest subjective image quality (score: 5; IQR: 4–5; *p* ≤ .001) and significant median increase (29%) in FOM (*p* < .001). Differences in detection were identified only for lesions ≤ 0.5 cm: 32/33 lesions were detected with DLIR_M and 26 lesions were detected with ASiR-V 50% (*p* = .031). Lesion accuracy of was 93.8% (95% CI: 88.1, 97.3; 122 of 130 lesions) for DLIR and 87.7% (95% CI: 80.8, 92.8; 114 of 130 lesions) for ASiR-V 50%.

**Conclusions:**

DLIR yields superior image quality and provides higher diagnostic accuracy compared to ASiR-V in the assessment of hypovascular liver lesions, in particular for lesions ≤ 0.5 cm.

**Clinical relevance statement:**

Deep learning image reconstruction algorithm demonstrates higher diagnostic accuracy compared to iterative reconstruction in the identification of hypovascular liver lesions, especially for lesions ≤ 0.5 cm.

**Key Points:**

*• Iterative reconstruction algorithm impacts image texture, with negative effects on diagnostic capabilities.*

*• Medium-strength deep learning image reconstruction algorithm outperforms iterative reconstruction in the diagnostic accuracy of ≤ 0.5 cm hypovascular liver lesions (93.9% vs 78.8%), also granting higher objective and subjective image quality.*

*• Deep learning image reconstruction algorithm can be safely implemented in routine abdominal CT protocols in place of iterative reconstruction.*

**Supplementary Information:**

The online version contains supplementary material available at 10.1007/s00330-023-10171-8.

## Introduction

Computed tomography (CT) is considered the reference standard for diagnosis, staging, and monitoring response to therapy of abdominal oncologic disease, owing to its fast execution, high availability, and consistent reproducibility [1]. Oncologic patients need to undergo strict follow-up consisting of multiple CT examinations [2]; in this scenario, it is crucial to minimize radiation dose and cumulative effective dose.

Filtered back-projection (FBP) has represented the conventional image reconstruction algorithm for over 30 years, owing to its good performances at standard radiation dose levels. However, increased awareness of radiation exposure along with soaring progresses in computational power paved the way for iterative reconstruction algorithms to replace FBP. Although this new technology is effective in reducing image noise and, consequently, in enabling low-dose CT examinations, many radiologists have complained about the “unnatural” and “unfamiliar” appearance of the images in clinical practice [3]. Steady rise in computing power enabled the implementation of deep learning image reconstruction (DLIR) algorithms, based on neural network models [4] and capable of learning from input data; DLIR exploits the capabilities of artificial intelligence to overcome IR limitations and further improve image quality. Preliminary studies [5–7] have proved DLIR algorithm effective in improving image quality without producing unnatural images at lower radiation doses in cardiovascular [8] and chest imaging [9] and in detecting abdominal lesions [10]. Recent studies evaluating the differences between DLIR and adaptive statistical iterative reconstruction (ASiR-V) showed that DLIR datasets acquired at low dose displayed improved image noise, signal-to-noise (SNR) ratio, and contrast-to-noise ratio (CNR) compared to iterative images at standard-dose CT, and were favored by most readers [6, 11–14]. However, to the best of our knowledge, a broad comparison of DLIR and ASiR-V at their respective full set of strength levels has not been reported yet.

Thus, the aim of our study was to perform a comprehensive within-subject image quality analysis of abdominal CT examinations reconstructed with DLIR and to evaluate diagnostic accuracy compared to the routinely applied ASiR-V algorithm.

## Materials and methods

### Study population

This prospective randomized study was conducted at Sant’Andrea University Hospital, Rome, Italy, and was approved by the Institutional Review Board. Written informed consent was obtained from all patients.

Consecutive oncologic patients [6, 11–14] were prospectively enrolled from September 2021 to January 2022. Exclusion criteria were as follows: pregnancy, age < 18 years, kidney failure (eGFR < 30 mL/min/1.73 m^2^), previous allergic reaction to iodinated contrast medium (CM), and severe motion artifacts on CT images impairing the qualitative and quantitative measurement. Data regarding patients’ age, gender, height, total body weight, lean body weight, body mass index (BMI), and radiation exposure were also recorded.

### CT image acquisition

All patients underwent CECT on a 128-slice CT (GE Revolution EVO CT Scanner, GE Medical Systems) in supine position at full inspiration, in cranio-caudal direction, before and after CM injection.

The volume of CM was calculated based on lean body weight, using the James formula [15]. Each patient was injected 0.7 g of iodine per kilogram of lean body weight, which was then divided by the concentration of CM, as follows:

$$\mathrm{CM\;volume}\;\left(\mathrm{mL}\right)=\frac{0.7 \cdot\mathrm{LBW}}{\mathrm{CM\;concentration}} \cdot1000$$

A non-ionic contrast medium (400 mgI/mL iomeprol, Iomeron 400; Bracco Imaging) was intravenously injected to all patients at a flow rate of 3/3.5 mL/s through an 18-gauge antecubital access, by means of a triple-syringe power injector (MEDRAD® Centargo CT Injection System; Bayer AG), chased by 50 mL of saline solution at corresponding flow rate.

Scan delay was set by a dedicated bolus-tracking technique application (SmartPrep, GE Healthcare), by placing a 120 HU threshold region of interest (ROI) within the abdominal aorta at the level of the celiac axis, a 15 s delay was used for the arterial phase, a 60 s delay was used for the portal venous phase, and a 180 s delay was used for the delayed phase.

Patients were scanned with a low-dose protocol with the following parameters: tube voltage of 80 kVp for the arterial phase and 100 kVp for the portal venous and delayed phases, automatic current modulation range 100–240 mA (Auto-mAs, GE Healthcare); detector collimator configuration 0.625 × 64 mm with *z*-flying focal spot technique; beam collimation 40 mm; pitch 0.984:1; dose reduction 50% (smart-mA, GE Healthcare); gantry speed 0.6 s.

### CT image reconstruction

Raw data were reconstructed at scan FOV: 50 cm and DFov: 34/36 cm (variable), utilizing standard abdominal kernel, matrix of 512*512, with a 1.250 mm slice spacing and thickness using two different algorithms: iterative reconstruction (ASiR-V; GE Healthcare) at strength levels from 10 to 100% with a 10% interval, and DLIR (TrueFidelity™, GE Healthcare) with three intensity levels of reconstruction (high, medium, and low); therefore, a total of thirteen datasets were generated for each examination.

### Objective image quality analysis

Objective image quality was evaluated in portal venous phase by a reader with 16 years of experience in abdominal imaging on a dedicated workstation (adw4.7, GE Healthcare), for each patient and in all reconstructed datasets. On axial slices, liver attenuation values (HU) were calculated by placing three circular ROIs of identical size (1cm^2^) in the hepatic segments IVb, V, and VI, avoiding intrahepatic vessel, and eventually averaged. Standard deviation (SD) of the ROI drawn in the left latissimus dorsi muscle was defined as image noise.

All ROIs were placed three times, and measurements have been averaged to minimize measurement inaccuracies. Consistency on ROI placement throughout the datasets was ensured by using the copy-paste tool of the workstation.

Signal-to-noise ratio (SNR) was calculated as follows:

$$\mathrm{SNR }= \frac{{\mathrm{HU}}_{\mathrm{liver}}}{\mathrm{noise}}$$

Contrast-to-noise ratio (CNR) was calculated as follows:

$$\mathrm{CNR }= \frac{{\mathrm{HU}}_{\mathrm{liver}}- {\mathrm{HU}}_{\mathrm{muscle}}}{\mathrm{noise}}$$

### Subjective image quality analysis

Subjective image quality analysis was performed by two readers with 12 and 10 years of experience in abdominal imaging, blinded to reconstruction protocol, on ASiR-V 50%, ASiR-V 100%, DLIR_M, and DLIR_H datasets, in consensus reading. The analysis was limited to these datasets based on results of objective image quality analysis (ASiR-V 100% and DLIR_H), routine clinical practice (ASiR-V 50%), and vendor recommendations (DLIR_M). Images were evaluated with standard window setting (width, 350 HU; level, 40 HU) but freely adjustable to suit readers’ preferences. Ambient light was kept constant at circa 35–40 lx.

To minimize recall bias, images were randomly assessed and no more than two different reconstructed datasets from each patient were analyzed during each interpretation, maintaining a time interval of 7 days between sessions.

Image quality was calculated using an ordinal 5-point Likert scale (1, uninterpretable examination; 2, poor; 3, acceptable; 4, good; and 5, excellent image quality) [16].

### Figure of merit

The dose-length product (DLP) of the arterial and delayed phase was annotated for each patient. The effective radiation dose (ED) was calculated for each patient by multiplying the DLP with a conversion factor *k* of 0.015 mSv·mGy^−1^ · cm^−1^ [16, 17].

Since acquisitions in the arterial phase and in the delayed were performed at different tube voltages (80 kV vs 100 kV, respectively), in order to evaluate differences in objective image quality independently of the ED [18], the SNR and figure of merit (FOM) of the latissimus dorsi muscle were calculated as follows.

$${\mathrm{SNR}}_{\mathrm{muscle}} = \frac{{\mathrm{HU}}_{\mathrm{muscle}}}{\mathrm{noise}}$$

$$\mathrm{FOM}= \frac{{{\mathrm{SNR}}_{\mathrm{muscle}}}^{2}}{\mathrm{DLP }\times 0.015}$$

Muscle was preferred over liver parenchyma due to its stable density measurement after contrast medium injection [19].

### Reference standard and lesion detection

The reference standard was assessed by three radiologists with 38, 27, and 26 years of experience in abdominal imaging, in consensus, using all clinical data and cross-sectional imaging examinations available at our institution; liver lesions were classified in a dichotomous fashion as benign or malignant. Benign lesions scored ≥ 3 on the malignancy scale were deemed false-positive; malignant lesions either scored ≤ 2 on the malignancy scale or not identified were considered false-negative [20].

Two board-certified radiologists, with 12 and 10 years of experience in abdominal radiology, respectively, performed lesion detection on the portal venous phase, blinded to patients’ information except cancer diagnosis. DLIR and ASIR-V datasets of each patient were assessed in a randomized order, in five sessions; to minimize recall biases, DLIR and ASiR-V of the same patient were always assessed in different sessions.

Hypoattenuating liver lesions measuring ≥ 2 mm were marked and characterized with a 5-point Likert scale (1, definitely benign; 2, likely benign; 3, malignancy not excluded; 4, likely malignant; 5, definitely malignant); diagnostic confidence was also assessed with a 5-point Likert scale (from 1: very low confidence to 5: very high confidence) [21].

### Radiation dose

The CTDI_vol_ and DLP were recorded for each examination; ED was eventually calculated as previously mentioned [16].

### Statistical analysis

Statistical analyses were performed by means of a dedicated software (IBM Corp. Released 2017. IBM SPSS Statistics for Macintosh, Version 25.0. IBM Corp). Normality of data distribution was assessed with the Kolmogorov–Smirnov test.

Continuous variables were expressed as mean ± SD or as median and interquartile range (IQR), according to their distribution; categorical variables were expressed as median and IQR.

Liver attenuation values, image noise, objective image quality, and lesion confidence score were compared using the repeated-measures ANOVA test or Friedman test, as appropriate. The Wilcoxon signed-rank test was conducted to assess the differences in FOM between DL_M and ASiR-50% reconstructions. Differences in subjective image quality among the different reconstruction datasets were assessed with the Kruskal–Wallis *H* test. Diagnostic accuracy differences between DLIR_M and ASiR-V 50% were assessed with the McNemar test. A *p*-value < 0.05 was considered to indicate a statistically significant result; Bonferroni correction was applied to adjust post hoc pairwise comparisons.

## Results

### Patient population

**Fig. 1:**
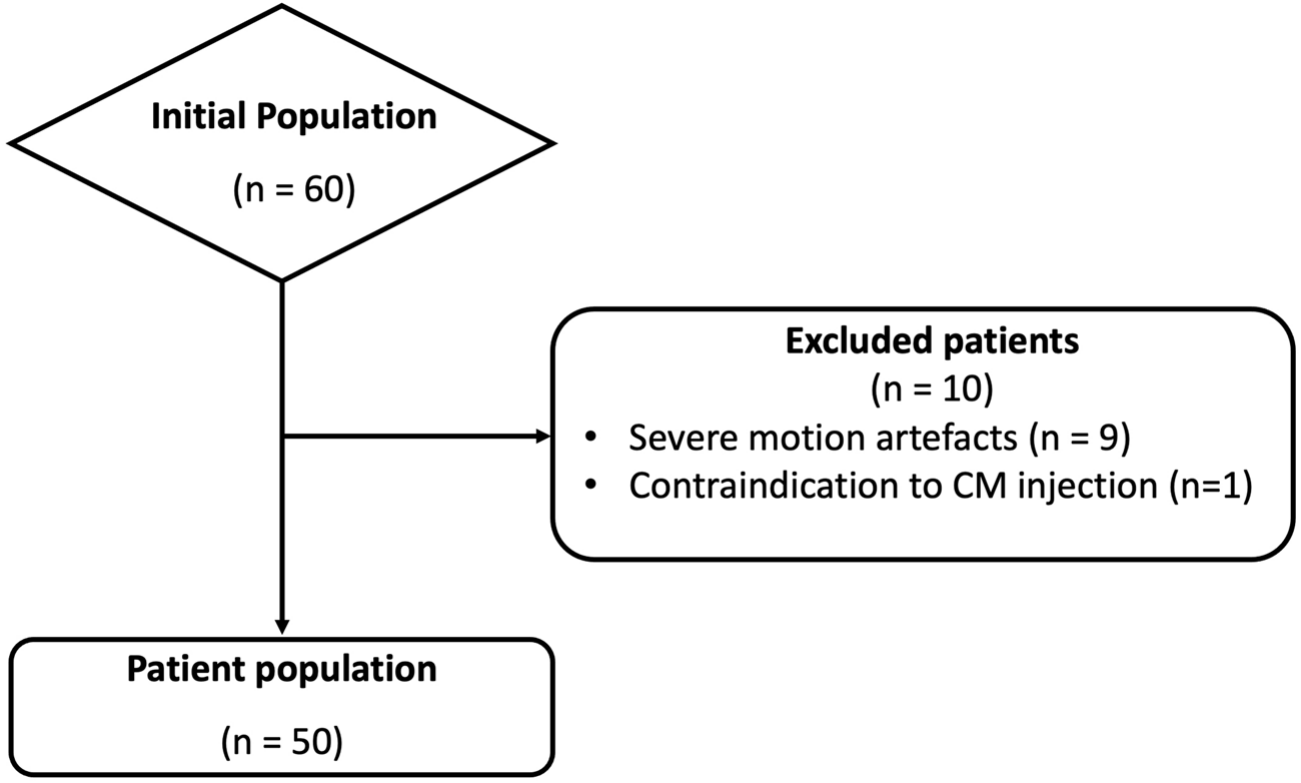
Flow diagram of patient recruitment

Patient characteristics are listed in Table 1, and patient flow diagram is displayed in Fig. 1. The final population consisted of 50 patients (27 females), with a mean age of 70 ± 10 years (range 47–87 years) and a mean BMI of 26.3 ± 5.4 kg/m^2^ (range 14.7–39.8 kg/m^2^). Reference standard assessment identified 130 liver lesions (105 benign lesions and 25 metastases).

**Table 1:** Patient characteristics

Parameter	Value
No. of patients	50
Age (years)*	70 ± 10 (47–87)
Male-to-female ratio	23:27
Body mass index (kg/m^2^)*	26.3 ± 5.4 (14.7–39.8)

^*^Data are mean ± standard deviation (range)

### Objective image quality

Full objective image quality scores are displayed in Table 2.

**Table 2:** Objective image quality scores of ASiR-V and DLIR reconstruction

	ASiR-V 10%	ASiR-V 20%	ASiR-V 30%	ASiR-V 40%	ASiR-V 50%	ASiR-V 60%	ASiR-V 70%	ASiR-V 80%	ASiR-V 90%	ASiR-V 100%	DLIR_L	DLIR_M	DLIR_H
Attenuation	122.9 ± 19.7	123.0 ± 18.6	123.1 ± 18.4	123.5 ± 19.1	123.1 ± 18.3	122.7 ± 19.0	123.0 ± 18.1	123.3 ± 18.0	123.7 ± 18.7	123.3 ± 18.1	125.0 ± 18.4	124.3 ± 18.3	124.3 ± 18.0
Noise	33.9 ± 5.8	30.4 ± 5.0	26.2 ± 4.3	24.5 ± 4.6	21.2 ± 3.5	19.3 ± 3.2	17.5 ± 2.9	15.5 ± 2.8	13.5 ± 2.7	11.9 ± 2.4	19.2 ± 3.8	15.0 ± 2.5	10.6 ± 1.8
SNR	3.71 ± 0.8	4.14 ± 0.9	4.80 ± 1.0	5.17 ± 1.1	5.92 ± 1.2	6.50 ± 1.3	7.21 ± 1.5	8.16 ± 1.7	9.50 ± 2.4	10.7 ± 2.6	6.74 ± 1.7	8.43 ± 1.8	11.9 ± 2.8
CNR	1.94 ± 0.6	2.13 ± 0.7	2.51 ± 0.8	2.72 ± 0.9	3.10 ± 1.0	3.40 ± 1.1	3.80 ± 1.1	4.32 ± 1.3	5.03 ± 1.6	5.69 ± 1.8	3.54 ± 1.1	4.42 ± 1.3	6.14 ± 1.7

*ASiR-V*, adaptive statistical iterative reconstruction algorithm; *CNR*, contrast-to-noise ratio; *DLIR*, deep learning image reconstruction algorithm; *SNR*, signal-to-noise ratio

Data are mean ± standard deviation

**Fig. 2:**
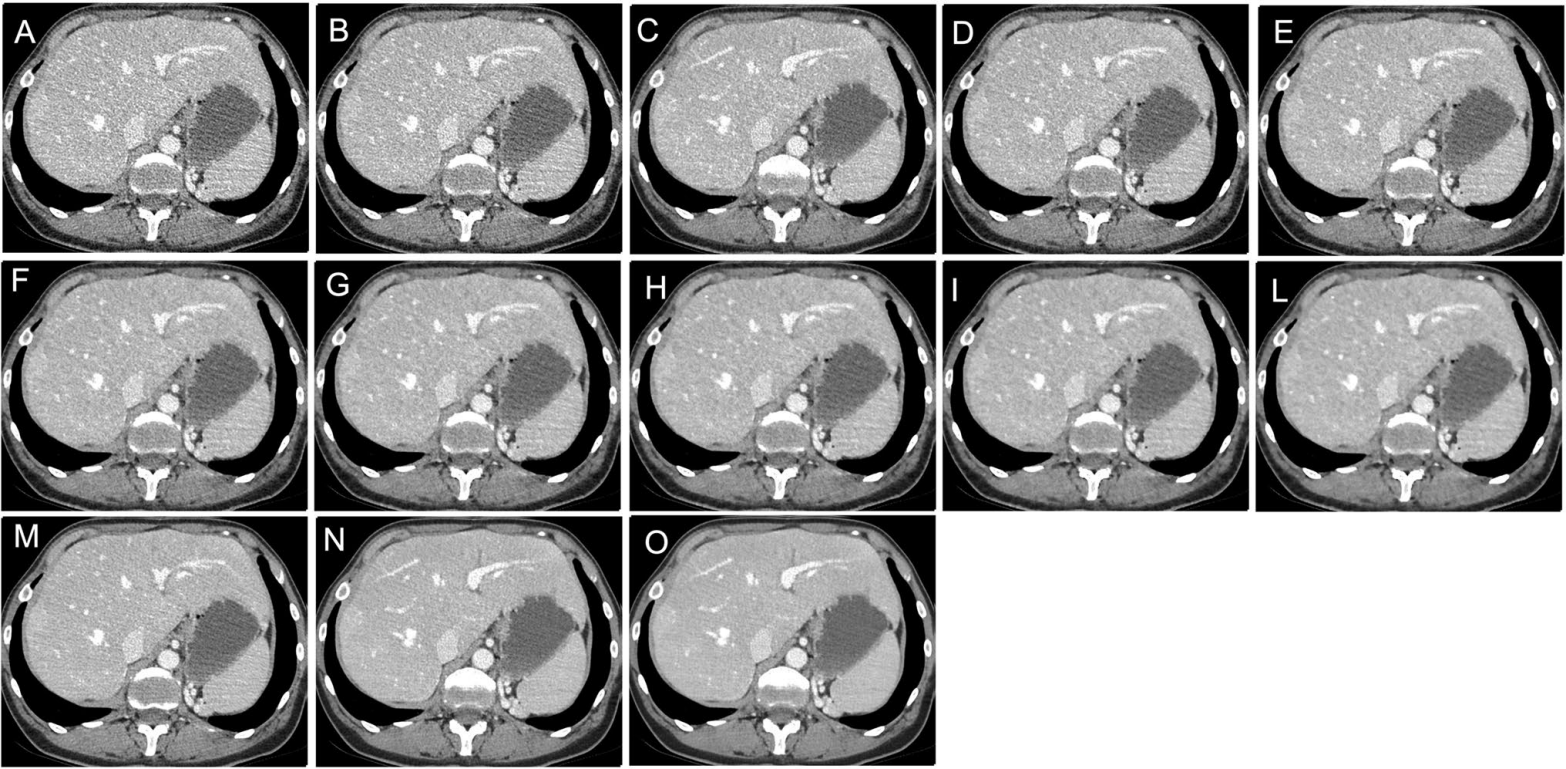
Axial CT images reconstructed with ASiR-V from 10 to 100%, with 10% intervals (**A** to **L**), and with DLIR at low (**M**), medium (**N**), and high (**O**) strength levels. ASiR-V, hybrid iterative reconstruction algorithm; DLIR, deep learning image reconstruction algorithm

A total of 650 datasets were analyzed. The lowest noise was obtained by DLIR_H (10.6 ± 1.8) significantly lower than ASiR-V 100% (11.9 ± 2.4; *p* = 0.043), DLIR_M (15.0 ± 2.5), DLIR_L (19.2 ± 3.8), and all the other ASiR-V datasets (all *p* < 0.001; Fig. 2).

The DLIR_M dataset showed comparable image noise with ASiR-V 80% (15.0 ± 2.5 vs 15.5 ± 2.8, respectively; *p* = 1), and lower noise than DLIR_L (19.2 ± 3.8; *p* < 0.001). The DLIR_L dataset exhibited comparable image noise with ASiR-V 60% (19.3 ± 3.2; *p* = 1) and ASiR-V 70% (17.5 ± 2.9; *p* = 0.155).

SNR peaked with DLIR_H (11.9 ± 2.8), resulting similar to ASiR-V 100% (10.7 ± 2.6; *p* = 0.051) and greater than DLIR_M (8.43 ± 1.8), DLIR_L (6.74 ± 1.7), and the other ASiR-V datasets (all *p* < 0.001). The DLIR_M dataset presented similar SNR with ASiR-V 80% (8.43 ± 1.8 vs 8.16 ± 1.7, respectively; *p* = 1), and greater SNR than DLIR_L (6.74 ± 1.7; *p* < 0.001). The DLIR_L dataset presented similar SNR with ASiR-V 60% and ASiR-V 70% (*p* = 1). Pairwise comparisons are displayed in Supplemental Table 1.

CNR peaked with DLIR_H (6.14 ± 1.7), resulting similar to ASiR-V 100% (5.69 ± 1.8; *p* = 1) and greater than DLIR_M (4.42 ± 1.3), DLIR_L (3.54 ± 1.1), and the other ASiR-V datasets (all *p* ≤ . 001). The DLIR_M dataset presented similar CNR with ASiR-V 80% (8.43 ± 1.8 vs 8.16 ± 1.7, respectively; *p* = 1) and greater CNR than DLIR_L (6.74 ± 1.7; *p* < 0.001). The DLIR_L dataset presented similar CNR with ASiR-V 60% and ASiR-V 70% (*p* = 1). Pairwise comparisons are displayed in Supplemental Table 2.

### Subjective image quality

**Fig. 3:**
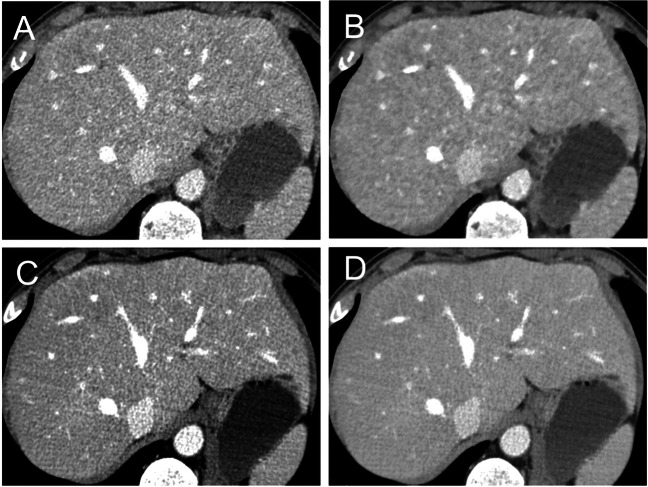
Axial CT images of a 57-year-old male reconstructed with ASiR-V 50% (**A**), ASiR-V 100% (**B**), DLIR_M (**C**), and DLIR_H (**D**). ASiR-V, hybrid iterative reconstruction algorithm; DLIR, deep learning image reconstruction algorithm

Full subjective image quality scores and pairwise comparisons are displayed in Table 3. No examination was judged uninterpretable (score: 1). DLIR_M obtained the greatest image quality (score: 5; IQR: 4–5), significantly greater than the remaining datasets (all *p* ≤ 0.001). No statistical differences were found between DLIR_H (score: 4; IQR: 3–5) and ASiR-V 50% (score: 4; IQR: 4–5; *p* = 0.63); the lowest score was obtained by the ASiR-V 100% dataset (score: 3; IQR: 3–4; all *p* ≤ 0.012; Fig. 3).

**Table 3:** Subjective image quality scores of ASiR-V and DLIR reconstructions, with related pairwise comparisons

		Pairwise comparisons			
	Score^†^	ASiR-V 50%	ASiR-V 100%	DLIR_M	DLIR_H
ASiR-V 50%	4 (4–4)		.012	.001	.063*
ASiR-V 100%	3 (3–4)	.012		< .001	.001
DLIR_M	5 (4–5)	.001	< .001		.001
DLIR_H	4 (3–4)	.063*	.001	.001	

*ASiR-V*, adaptive statistical iterative reconstruction algorithm; *DLIR*, deep learning image reconstruction algorithm

^†^Data are median (interquartile range)

^*^Non-statistically significant *p*-values

### Figure of merit

The arterial phase reconstructed with DLIR_M had a significantly lower DLP than the delayed phase reconstructed with ASiR-V 50% (150.4 vs 212.4; *p* < 0.05). FOM of DLIR_M was higher than of ASiR-V 50% in 39/50 (78%) datasets, and lower in 11/50 (22%) cases. Overall, there was statistically significant median increase in FOM (29%) with DLIR_M reconstruction (median: 3.744; IQR: 2.098–6.505 vs median: 2.634; IQR: 1.636–4.254; *z* = 4.33; *p* < 0.001).

### Diagnostic accuracy

**Fig. 4:**
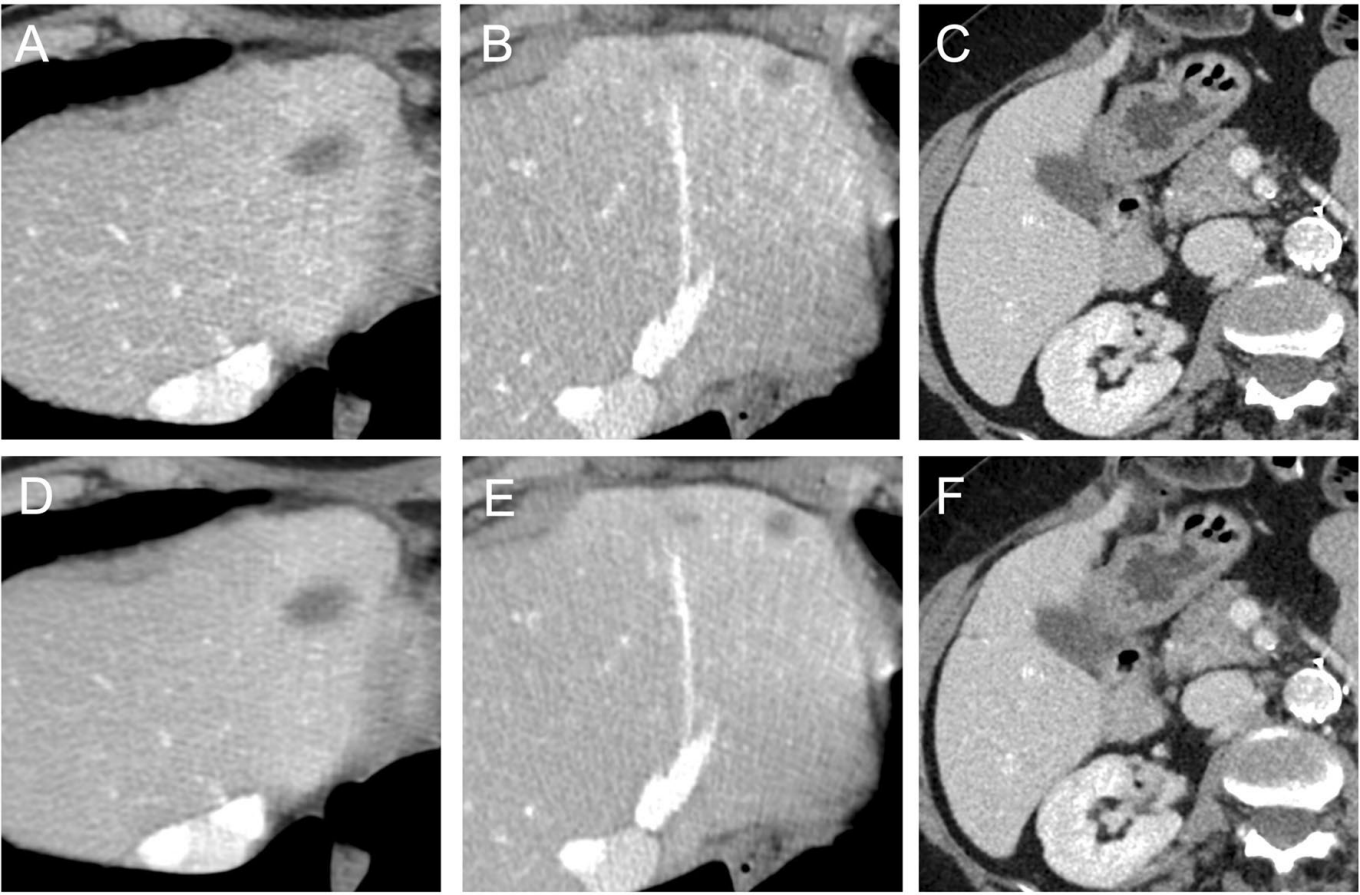
Axial CT images of a 54-year-old woman showing liver lesions > 10 mm, 6–10 mm, and ≤ 5 mm reconstructed with ASiR-V 50% (**A**, **B**, and **C**, respectively) and DLIR_M (**D**, **E**, and **F**). ASiR-V, hybrid iterative reconstruction algorithm; DLIR, deep learning image reconstruction algorithm

Comprehensive data are displayed in Table 4. Overall, 127 of 130 lesions were detected with DLIR (97.7%; 95% CI: 93.4, 99.5) and 120 of 130 lesions were detected with ASiR-V (92.3%; 95% CI: 86.3, 96.3) (*p* = 0.016); in particular, DLIR detected a significantly higher number of small lesions compared to ASiR-V (*p* = 0.041; Fig. 4). Lesion confidence score was higher for DLIR (median: 5; IQR: 5–5) compared to that for ASiR-V (median: 5; IQR: 4–5) (*p* < 0.001). The overall lesion characterization accuracies were 93.8% (95% CI: 88.2, 97.3; 122 of 130 lesions) for DLIR and 87.7% (95% CI: 80.8, 92.8; 114 of 130 lesions) for ASiR-V (*p* = 0.039). The overall sensitivities were 92.3% (95% CI: 74.9, 99.1; 24 of 26 lesions) for DLIR and 70.6% (95% CI: 52.5, 84.9; 24 of 34 lesions) for ASiR-V; the overall specificities were 95.1% (95% CI: 89.0, 98.4; 98 of 103) for DLIR and 93.8% (95% CI: 86.9, 97.7; 90 of 96) for ASiR-V.

**Table 4:** Diagnostic accuracy

	DLIR			ASiR-V		
	≤ 5 mm	6–10 mm	> 10 mm	≤ 5 mm	6–10 mm	> 10 mm
Detection (%)	97.0 (84.2, 99.9) [32/33]	96.0 (86.0, 99.5) [47/49]	100 (92.6, 100) [48/48]	78.8 (61.1, 91.0) [26/33]	93.9 (83.1, 98.7) [46/49]	100 (92.6, 100) [48/48]
Sensitivity (%)	50.0 (1.3, 98.7) [1/2]	84.6 (54.6, 98.1) [11/13]	100 (73.3, 100) [12/12]	12.5 (0.3, 52.7) [1/8]	78.6 (49.2, 95.3) [11/14]	100 (73.3, 100) [12/12]
Specificity (%)	96.8 (83.3, 99.9) [30/31]	94.4 (81.3, 99.3) [34/36]	94.4 (81.3, 99.3) [34/36]	100 (86.3, 100) [25/25]	88.6 (73.3, 96.8) [31/35]	94.4 (81.3, 99.3) [34/36]
Accuracy (%)	93.9 (79.8, 99.3) [31/33]	91.8 (80.4, 97.7) [45/49]	94.4 (85.8, 99.5) [46/48]	78.8 (61.1, 91.0) [26/33]	85.7 (72.8, 94.1) [42/49]	94.4 (85.8, 99.5) [46/48]

Performance data are per lesion

Numbers in parentheses are 95% CIs; numbers in brackets are numbers of lesions

*ASiR-V 50%*, adaptive statistical iterative reconstruction algorithm; *DLIR*, deep learning image reconstruction algorithm

### Radiation dose

The mean CTDI_vol_ and DLP were 24.1 ± 8.6 mGy and 786.3 ± 291.7 mGy cm, for an estimated mean ED of 11.8 ± 4.4 mSv.

## Discussion

Our investigation demonstrates that DLIR at medium strength improves liver lesion detection rate compared to ASiR-V 50% (*p* = 0.016). While the two algorithms detected a comparable number of lesions larger than 0.5 cm, DLIR outperformed ASiR-V in the detection of liver lesions smaller than 0.5 cm (*p* = 0.031). Additionally, DLIR obtained a higher overall diagnostic accuracy (*p* = 0.039) and a higher lesion confidence score (*p* < 0.001) compared to ASiR-V 50%. Along with better diagnostic performance, our investigation documented higher objective and subjective image quality of DLIR compared to ASiR-V: while DLIR at high strength achieved the highest SNR and CNR, DLIR at medium strength obtained the highest subjective quality score.

Full exploitation of iterative reconstruction algorithms is hampered by their detrimental effect on image texture, especially at high strength levels, resulting in the generation oversmoothed images [22, 23], which ultimately might have a negative effect on diagnostic capabilities. On the contrary, DLIR algorithm does not have a detrimental impact on image texture [7], returning higher objective image quality at same radiation dose levels and comparable image quality when used to reconstruct low-dose CT acquisitions. As a result, DLIR is now under current investigation in different clinical settings, outperforming IR in terms of image noise and image quality in abdominal [14, 24, 25], cardiac [8], and chest imaging [6, 9, 26, 27]; focusing on abdominal imaging, its higher performance compared to IR in terms of image quality and lesion conspicuity has been also demonstrated in the setting of dual-energy CT [28]. Therefore, its implementation in clinical practice is constantly growing, preluding a gradual replacement of iterative reconstruction algorithms [13].

Our investigation demonstrated DLIR is effective in achieving a significantly higher FOM compared to ASiR-V, despite a 29% lower radiation dose. The possibility of sensibly lowering radiation exposure without sacrificing the diagnostic yield of a CT examination is strictly related to the specific clinical task [29, 30], and abdominal studies are typically quite sensible to radiation dose due to the intrinsic low contrast differences between different abdominal organs. In particular, a high image quality is mandatory in liver imaging in order to identify and adequately characterize liver lesions, especially small ones, whose evaluation might be compromised by modest radiation dose reduction not counterbalanced by iterative reconstruction algorithms [31]. In this regard, DLIR, already proven effective in maintaining noise texture and adequate low contrast liver lesion detectability at low-dose settings [32], might enable further dose optimization in abdominal CT with no detrimental impact on diagnostic performances [33].

Jensen et al demonstrated that DLIR applied to reduced-dose CT preserved detection of liver lesions larger than 0.5 cm when compared to standard-dose CT reconstructed with FBP, while the latter outperformed DLIR in detecting smaller lesions [20]. Our investigation transfers these results to iterative reconstruction, demonstrating similar performance between DLIR and ASiR-V in the detection of lesion larger than 0.5 cm. On the other hand, our findings demonstrated that DLIR outperformed ASiR-V in the detection of lesions smaller than 0.5 cm. These differences might be explained by differences in study design, since our investigation compared the two reconstruction algorithms in the same CT acquisition.

Clinical implications of such findings indicate that DLIR can be safely implemented in routinely used clinical protocols in place of iterative reconstruction algorithms. On the contrary, particular attention should be paid to the design of dedicated low-dose DLIR CT protocols, since the benefits of a low dose burden might not be sustained by adequate diagnostic performance on the new algorithm in the detection of small liver lesions, making it unsuitable in clinical practice. Hence, large prospective trials should be performed in order to establish adequate and robust low-dose scan protocol clinically suitable for DLIR reconstruction.

Our investigation should be evaluated in light of some limitation. First, despite the study population was formed by oncologic patients, the characterization of liver lesions was based on clinical data and cross-sectional imaging examination; nevertheless, the creation of a consensus-based reference standard is robust and consistent with earlier examinations [20, 31]. Second, the sample size is relatively small and further studies with a larger number of participants are highly advisable to strengthen and expand upon our results. Third, this investigation analyzed the performances of a single vendor algorithm, specifically in liver parenchyma; therefore, our results might not be directly applicable to other vendors and in different body regions; investigations comparing the diagnostic performance of different DLIR algorithms might be indeed of great interest.

In conclusion, DLIR yields superior image quality and provides higher diagnostic accuracy compared to ASiR-V in the assessment of hypovascular liver lesions, in particular for lesions smaller than 0.5 cm. These higher diagnostic performances allow the design of low-dose acquisition protocols able to maintain current diagnostic accuracy with lower radiation burden. Nevertheless, further investigations are needed to establish appropriate radiation dose levels based on specific clinical tasks, avoiding detrimental effect of excessive dose reduction not compensated by DLIR denoising capabilities.

### Supplementary Information

Below is the link to the electronic supplementary material.

Supplementary file1 (PDF 200 KB)

## Abbreviations

ASiR-V: Adaptive statistical iterative reconstruction
BMI: Body mass index
CM: Contrast medium
CNR: Contrast-to-noise ratio
DLIR: Deep learning image reconstruction
DLP: Dose-length product
ED: Effective dose
FBP: Filtered back-projection
FOM: Figure of merit
ROI: Region-of-interest
SNR: Signal-to-noise ratio

## Declarations

### Guarantor

The scientific guarantor of this publication is Andrea Laghi.

### Statistics and biometry

No complex statistical methods were necessary for this paper.

### Study subjects or cohorts overlap

No study or cohorts have been previously reported.

### Methodology

• prospective

• experimental

• performed at one institution
