# Supplementary material for: Low-dose liver CT: image quality and diagnostic accuracy of deep learning image reconstruction algorithm
Source: Eur Radiol. 2023 Sep 9;34(4):2384–93. doi: 10.1007/s00330-023-10171-8 (PMC10957592; doi:10.1007/s00330-023-10171-8)
Supplement: Supplementary file 1 — Supplementary file1 (PDF 200 KB) [file 330_2023_10171_MOESM1_ESM.pdf]

Supplemental Material

Supplemental Table 1: Pairwise comparisons of SNR achieved by FBP, ASiR-V, and DLIR reconstruction.

	ASiR-V 10%	ASiR-V 20%	ASiR-V 30%	ASiR-V 40%	ASiR-V 50%	ASiR-V 60%	ASiR-V 70%	ASiR-V 80%	ASiR-V 90%	ASiR-V 100%	DLIR_L	DLIR_M	DLIR_H
**ASiR-V 10%**		< .001	< .001	< .001	< .001	< .001	< .001	< .001	< .001	< .001	< .001	< .001	< .001
**ASiR-V 20%**	< .001		< .001	< .001	< .001	< .001	< .001	< .001	< .001	< .001	< .001	< .001	< .001
**ASiR-V 30%**	< .001	< .001		< .017	< .001	< .001	< .001	< .001	< .001	< .001	< .001	< .001	< .001
**ASiR-V 40%**	< .001	< .001	< .017		< .001	< .001	< .001	< .001	< .001	< .001	< .001	< .001	< .001
**ASiR-V 50%**	< .001	< .001	< .001	< .001		< .001	< .001	< .001	< .001	< .001	< .005	< .001	< .001
**ASiR-V 60%**	< .001	< .001	< .001	< .001	< .001		< .001	< .001	< .001	< .001	1*	< .001	< .001
**ASiR-V 70%**	< .001	< .001	< .001	< .001	< .001	< .001		< .001	< .001	< .001	1*	< .001	< .001
**ASiR-V 80%**	< .001	< .001	< .001	< .001	< .001	< .001	< .001		< .001	< .001	< .001	1*	< .001
**ASiR-V 90%**	< .001	< .001	< .001	< .001	< .001	< .001	< .001	< .001		< .001	< .001	.010	< .001
**ASiR-V 100%**	< .001	< .001	< .001	< .001	< .001	< .001	< .001	< .001	< .001		< .001	< .001	.051*
**DLIR_L**	< .001	< .001	< .001	< .001	.005	1*	1*	< .001	< .001	< .001		< .001	< .001
**DLIR_M**	< .001	< .001	< .001	< .001	< .001	< .001	< .001	1*	< .010	< .001	< .001		< .001
**DLIR_H**	< .001	< .001	< .001	< .001	< .001	< .001	< .001	< .001	< .001	.051*	< .001	< .001	

ASiR-V, adaptive statistical iterative reconstruction algorithm; DLIR, deep learning image reconstruction algorithm
* non-statistically significant *P*-values

Supplemental Table 2: Pairwise comparisons of CNR achieved by FBP, ASiR-V, and DLIR reconstruction.

	ASiR-V 10%	ASiR-V 20%	ASiR-V 30%	ASiR-V 40%	ASiR-V 50%	ASiR-V 60%	ASiR-V 70%	ASiR-V 80%	ASiR-V 90%	ASiR-V 100%	DLIR_L	DLIR_M	DLIR_H
**ASiR-V 10%**		.654*	< .001	< .001	< .001	< .001	< .001	< .001	< .001	< .001	< .001	< .001	< .001
**ASiR-V 20%**	.654*		< .001	< .001	< .001	< .001	< .001	< .001	< .001	< .001	< .001	< .001	< .001
**ASiR-V 30%**	< .001	< .001		.078*	< .001	< .001	< .001	< .001	< .001	< .001	< .001	< .001	< .001
**ASiR-V 40%**	< .001	< .001	.078*		< .001	< .001	< .001	< .001	< .001	< .001	< .001	< .001	< .001
**ASiR-V 50%**	< .001	< .001	< .001	< .001		< .001	< .001	< .001	< .001	< .001	.003	< .001	< .001
**ASiR-V 60%**	< .001	< .001	< .001	< .001	< .001		< .001	< .001	< .001	< .001	1*	< .001	< .001
**ASiR-V 70%**	< .001	< .001	< .001	< .001	< .001	< .001		< .001	< .001	< .001	1*	.001	< .001
**ASiR-V 80%**	< .001	< .001	< .001	< .001	< .001	< .001	< .001		< .001	< .001	< .001	1*	< .001
**ASiR-V 90%**	< .001	< .001	< .001	< .001	< .001	< .001	< .001	< .001		< .001	< .001	.015	< .001
**ASiR-V 100%**	< .001	< .001	< .001	< .001	< .001	< .001	< .001	< .001	< .001		< .001	< .001	1*
**DLIR_L**	< .001	< .001	< .001	< .001	.003	1*	1*	< .001	< .001	< .001		< .001	< .001
**DLIR_M**	< .001	< .001	< .001	< .001	< .001	< .001	.001	1*	.015	< .001	< .001		< .001
**DLIR_H**	< .001	< .001	< .001	< .001	< .001	< .001	< .001	< .001	< .001	1*	< .001	< .001	

ASiR-V, adaptive statistical iterative reconstruction algorithm; DLIR, deep learning image reconstruction algorithm
* non-statistically significant *P*-values
